# *catena*-Poly[[[triaqua­(nitrato-κ^2^ *O*,*O*′)neodymium(III)]-bis­(μ_2_-pyridinium-4-carboxyl­ato-κ^2^ *O*:*O*′)] bis­(perchlorate) monohydrate]

**DOI:** 10.1107/S1600536808043997

**Published:** 2009-01-08

**Authors:** Jia-Zhi Pu

**Affiliations:** a Department of Pharmacy, Zunyi Medical College, Zunyi, Guizhou 563003, People’s Republic of China

## Abstract

In the title compound, {[Nd(NO_3_)(C_6_H_5_NO_2_)_2_(H_2_O)_3_](ClO_4_)_2_·H_2_O}_*n*_, the Nd^III^ atom is nine-coordinated by four O atoms from four pyridinium-4-carboxyl­ate ligands, two O atoms from a chelating nitrate anion and three water mol­ecules in a distorted tricapped trigonal–prismatic coordination geometry. Adjacent Nd atoms are linked by the bidentate pyridinium-4-carboxyl­ate ligands into a chain running along the *b* axis. The chains are further connected by O—H⋯O and N—H⋯O hydrogen bonds into a three-dimensional network.

## Related literature

For related structures, see: Liao *et al.* (2004 ▶); Wang *et al.* (2004 ▶).
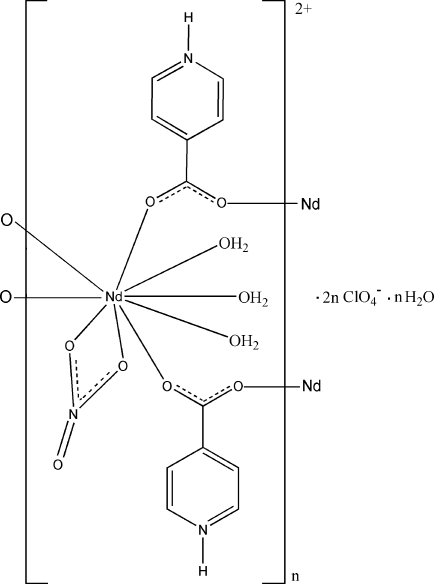


## Experimental

### 

#### Crystal data

[Nd(NO_3_)(C_6_H_5_NO_2_)_2_(H_2_O)_3_](ClO_4_)_2_·H_2_O
*M* *_r_* = 723.43
Triclinic, 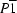

*a* = 8.3962 (7) Å
*b* = 10.1119 (8) Å
*c* = 14.7229 (12) Å
α = 81.663 (1)°
β = 79.601 (1)°
γ = 71.334 (1)°
*V* = 1159.68 (16) Å^3^
*Z* = 2
Mo *K*α radiation
μ = 2.57 mm^−1^
*T* = 273 (2) K
0.32 × 0.26 × 0.20 mm

#### Data collection

Bruker APEXII CCD diffractometer
Absorption correction: multi-scan (*SADABS*; Sheldrick, 1996 ▶) *T*_min_ = 0.459, *T*_max_ = 0.605
5967 measured reflections
4073 independent reflections
3923 reflections with *I* > 2σ(*I*)
*R*_int_ = 0.017

#### Refinement

*R*[*F* ^2^ > 2σ(*F* ^2^)] = 0.029
*wR*(*F* ^2^) = 0.077
*S* = 0.99
4073 reflections
334 parameters
12 restraints
H-atom parameters constrained
Δρ_max_ = 1.00 e Å^−3^
Δρ_min_ = −1.11 e Å^−3^

### 

Data collection: *APEX2* (Bruker, 2007 ▶); cell refinement: *SAINT* (Bruker, 2007 ▶); data reduction: *SAINT*; program(s) used to solve structure: *SHELXS97* (Sheldrick, 2008 ▶); program(s) used to refine structure: *SHELXL97* (Sheldrick, 2008 ▶); molecular graphics: *SHELXTL* (Sheldrick, 2008 ▶); software used to prepare material for publication: *SHELXTL*.

## Supplementary Material

Crystal structure: contains datablocks I, global. DOI: 10.1107/S1600536808043997/hy2175sup1.cif

Structure factors: contains datablocks I. DOI: 10.1107/S1600536808043997/hy2175Isup2.hkl

Additional supplementary materials:  crystallographic information; 3D view; checkCIF report

## Figures and Tables

**Table 1:** Selected bond lengths (Å)

Nd1—O1	2.428 (3)
Nd1—O2^i^	2.392 (3)
Nd1—O3	2.446 (3)
Nd1—O4	2.571 (3)
Nd1—O5	2.651 (3)
Nd1—O7^ii^	2.390 (3)
Nd1—O1*W*	2.542 (3)
Nd1—O2*W*	2.567 (3)
Nd1—O3*W*	2.505 (3)

Symmetry codes: (i) 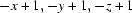; (ii) 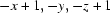.

**Table 2:** Hydrogen-bond geometry (Å, °)

*D*—H⋯*A*	*D*—H	H⋯*A*	*D*⋯*A*	*D*—H⋯*A*
O4*W*—H7*W*⋯O12	0.84	2.59	3.256 (11)	136
O4*W*—H8*W*⋯O5^i^	0.84	2.52	3.023 (8)	119
O4*W*—H8*W*⋯O8^i^	0.84	2.04	2.801 (9)	150
O3*W*—H5*W*⋯O12^ii^	0.84	2.11	2.945 (7)	170
O3*W*—H6*W*⋯O8	0.84	2.04	2.846 (8)	162
O2*W*—H3*W*⋯O1*W*^ii^	0.84	2.12	2.900 (5)	154
O2*W*—H4*W*⋯O4^iii^	0.84	2.04	2.861 (4)	167
O1*W*—H2*W*⋯O4*W*	0.84	1.83	2.593 (7)	150
O1*W*—H1*W*⋯O6^iv^	0.84	2.04	2.880 (5)	178
N2—H2*A*⋯O9^iii^	0.86	2.51	3.045 (5)	121
N2—H2*A*⋯O13^v^	0.86	2.48	3.033 (5)	123
N2—H2*A*⋯O10^vi^	0.86	2.15	2.868 (5)	141
N1—H1⋯O13^vii^	0.86	2.46	2.994 (6)	121
N1—H1⋯O9^viii^	0.86	2.46	2.988 (6)	120
N1—H1⋯O14^ix^	0.86	2.24	2.953 (6)	140

Symmetry codes: (i) 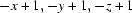; (ii) 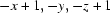; (iii) 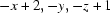; (iv) 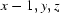; (v) 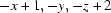; (vi) 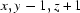; (vii) 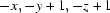; (viii) 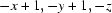; (ix) 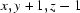.

**Fig. 1.**
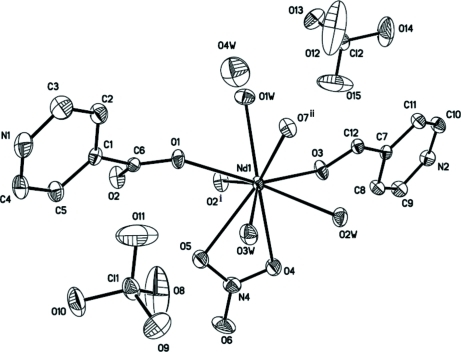
The asymmetric unit of the title compound, extended to show the Nd coordination. Displacement ellipsoids are drawn at the 30% probability level. H atoms have been omitted for clarity. [Symmetry codes: (i) 1 - x, 1 - y, 1 - z; (ii) 1 - x, -y, 1 - z.]

**Fig. 2.**
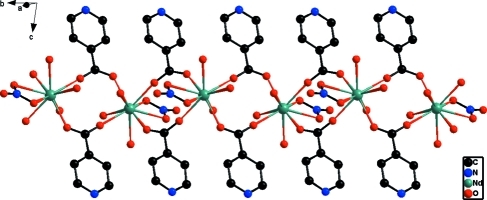
View of the polymeric chain. H atoms, perchlorate anions and uncoordinated water molecules are not shown for clarity.

**Fig. 3.**
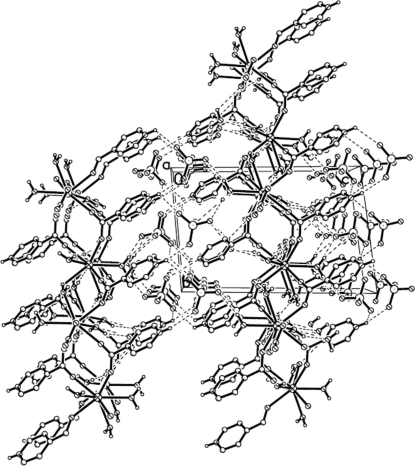
A packing view of the title compound, showing hydrogen bonds (dashed lines).

## supplementary crystallographic information

### Comment

In the structural investigation of isonicotinate complexes, it has been found that the isonicotinate functions as a multidentate ligand with versatile binding and coordination modes (Liao *et al.*, 2004; Wang *et al.*, 2004). In this paper, we report the crystal structure of the title compound, a new Nd^III^ complex resulted from the hydrothermal treatment of isonicotinic acid, Nd_2_O_3_ and a little nitric acid.

As depicted in Fig. 1, the asymmetric unit consists of one Nd^III^ atom, two pyridinium-4-carboxylate (Hint) ligands, one coordinated nitrate anion, three coordinated water molecules, two perchlorate anions and one uncoordinated water molecule. The Nd^III^ atom is nine-coordinated in a distorted tricapped trigonal prismatic coordination geometry, defined by four O atoms from four different Hint ligands, two O atoms from a nitrate anion and three water molecules (Table 1). The Hint ligands link the metal centres to form a polymeric chain (Fig. 2), in which the Nd^III^ atoms are separated by 5.586 (2) and 5.281 (3) Å. The chains are further self-assembled into a three-dimensional supramolecular network through O—H···O and N—H···O hydrogen bonds (Table 2 and Fig. 3).

### Experimental

A mixture of Nd_2_O_3_ (0.168 g, 0.5 mmol), isonicotinic acid (0.123 g, 1 mmol), HNO_3_ (0.12 ml) and H_2_O (10 ml) was placed in a 23 ml Teflon-lined reactor, which was heated to 433 K for 3 d and then cooled to room temperature at a rate of 10 K h^-1^. The crystals obtained were washed with water and dried in air.

### Refinement

H atoms on C and N atoms were positioned geometrically and treated as riding atoms, with C—H = 0.93 Å and N—H = 0.86 Å and with *U*_iso_(H) = 1.2*U*_eq_(C,*N*). H atoms of water molecules were located in difference Fourier maps and fixed in refinements, with O—H = 0.84 Å and *U*_iso_(H) = 1.5*U*_eq_(O).

### Crystal data

**Table:**

[Nd(NO_3_)(C_6_H_5_NO_2_)_2_(H_2_O)_3_](ClO_4_)_2_·H_2_O	*Z* = 2
*M**_r_* = 723.43	*F*(000) = 714
Triclinic, *P*1	*D*_x_ = 2.072 Mg m^−^^3^
Hall symbol: -P 1	Mo *K*α radiation, λ = 0.71073 Å
*a* = 8.3962 (7) Å	Cell parameters from 8000 reflections
*b* = 10.1119 (8) Å	θ = 1.7–26.0°
*c* = 14.7229 (12) Å	µ = 2.57 mm^−^^1^
α = 81.663 (1)°	*T* = 273 K
β = 79.601 (1)°	Block, colourless
γ = 71.334 (1)°	0.32 × 0.26 × 0.20 mm
*V* = 1159.68 (16) Å^3^	

### Data collection

**Table:**

Bruker APEXII CCD diffractometer	4073 independent reflections
Radiation source: fine-focus sealed tube	3923 reflections with *I* > 2σ(*I*)
graphite	*R*_int_ = 0.017
φ and ω scans	θ_max_ = 25.2°, θ_min_ = 2.1°
Absorption correction: multi-scan (*SADABS*; Sheldrick, 1996)	*h* = −7→10
*T*_min_ = 0.459, *T*_max_ = 0.605	*k* = −11→12
5967 measured reflections	*l* = −16→17

### Refinement

**Table:**

Refinement on *F*^2^	Primary atom site location: structure-invariant direct methods
Least-squares matrix: full	Secondary atom site location: difference Fourier map
*R*[*F*^2^ > 2σ(*F*^2^)] = 0.029	Hydrogen site location: inferred from neighbouring sites
*wR*(*F*^2^) = 0.077	H-atom parameters constrained
*S* = 0.99	*w* = 1/[σ^2^(*F*_o_^2^) + (0.0448*P*)^2^ + 3.0364*P*] where *P* = (*F*_o_^2^ + 2*F*_c_^2^)/3
4073 reflections	(Δ/σ)_max_ = 0.001
334 parameters	Δρ_max_ = 1.00 e Å^−^^3^
12 restraints	Δρ_min_ = −1.11 e Å^−^^3^

### Fractional atomic coordinates and isotropic or equivalent isotropic displacement parameters (Å^2^)

**Table:**

	*x*	*y*	*z*	*U*_iso_*/*U*_eq_	
C1	0.3296 (5)	0.6208 (4)	0.2806 (3)	0.0285 (8)	
Cl1	0.75800 (16)	0.47280 (12)	0.09482 (8)	0.0430 (3)	
N1	0.2205 (6)	0.7323 (4)	0.1159 (3)	0.0493 (11)	
H1	0.1879	0.7654	0.0628	0.059*	
Nd1	0.61497 (2)	0.219206 (19)	0.462773 (12)	0.02175 (9)	
O1	0.4362 (4)	0.4217 (3)	0.3801 (2)	0.0329 (6)	
C2	0.2041 (6)	0.5822 (5)	0.2522 (3)	0.0432 (11)	
H2	0.1574	0.5168	0.2886	0.052*	
Cl2	0.24041 (16)	0.02494 (12)	0.89509 (8)	0.0416 (3)	
N2	0.7879 (5)	−0.2315 (4)	0.8926 (2)	0.0364 (8)	
H2A	0.8157	−0.2643	0.9467	0.044*	
O2	0.4255 (4)	0.6291 (3)	0.4190 (2)	0.0406 (7)	
C3	0.1495 (7)	0.6428 (6)	0.1687 (4)	0.0557 (14)	
H3	0.0624	0.6207	0.1494	0.067*	
O3	0.6604 (4)	0.0616 (3)	0.6047 (2)	0.0374 (7)	
C4	0.3394 (7)	0.7728 (5)	0.1414 (3)	0.0440 (11)	
H4	0.3859	0.8362	0.1025	0.053*	
N4	0.9277 (5)	0.3174 (4)	0.4383 (3)	0.0388 (9)	
O4	0.9213 (4)	0.1992 (3)	0.4802 (3)	0.0451 (8)	
C5	0.3937 (6)	0.7207 (5)	0.2256 (3)	0.0380 (10)	
H5	0.4729	0.7523	0.2456	0.046*	
O5	0.7969 (4)	0.3949 (3)	0.4069 (2)	0.0455 (8)	
C6	0.4021 (5)	0.5516 (4)	0.3681 (3)	0.0247 (8)	
O6	1.0567 (5)	0.3518 (5)	0.4292 (3)	0.0688 (12)	
C7	0.6913 (5)	−0.1202 (4)	0.7252 (3)	0.0246 (8)	
O7	0.5371 (4)	−0.1017 (3)	0.6025 (2)	0.0350 (7)	
C8	0.8138 (6)	−0.0818 (5)	0.7579 (3)	0.0336 (9)	
H8	0.8628	−0.0169	0.7227	0.040*	
O8	0.8174 (13)	0.4411 (6)	0.1813 (4)	0.152 (4)	
C9	0.8617 (6)	−0.1400 (5)	0.8421 (3)	0.0380 (10)	
H9	0.9450	−0.1164	0.8643	0.046*	
O9	0.8797 (5)	0.3853 (5)	0.0322 (3)	0.0626 (11)	
C10	0.6734 (6)	−0.2736 (5)	0.8629 (3)	0.0376 (10)	
H10	0.6267	−0.3387	0.8995	0.045*	
O10	0.7386 (6)	0.6160 (4)	0.0700 (3)	0.0720 (13)	
C11	0.6248 (6)	−0.2203 (4)	0.7777 (3)	0.0329 (9)	
H11	0.5475	−0.2513	0.7553	0.039*	
O11	0.6059 (8)	0.4456 (7)	0.1061 (8)	0.177 (5)	
C12	0.6247 (5)	−0.0482 (4)	0.6362 (3)	0.0256 (8)	
O12	0.1848 (15)	0.0322 (7)	0.8114 (4)	0.197 (5)	
O13	0.1153 (5)	0.1236 (5)	0.9500 (3)	0.0634 (11)	
O14	0.2627 (8)	−0.1133 (5)	0.9314 (4)	0.108 (2)	
O15	0.3841 (9)	0.0555 (9)	0.8782 (10)	0.256 (8)	
O1W	0.3147 (4)	0.2628 (4)	0.5492 (2)	0.0426 (7)	
H1W	0.2375	0.2900	0.5154	0.064*	
H2W	0.2927	0.3121	0.5938	0.064*	
O2W	0.8124 (4)	−0.0227 (3)	0.4206 (2)	0.0397 (7)	
H4W	0.8961	−0.0633	0.4488	0.060*	
H3W	0.7594	−0.0782	0.4151	0.060*	
O3W	0.7472 (4)	0.2088 (3)	0.2962 (2)	0.0430 (8)	
H6W	0.7505	0.2762	0.2560	0.065*	
H5W	0.7699	0.1337	0.2717	0.065*	
O4W	0.2124 (9)	0.3371 (7)	0.7164 (4)	0.123 (2)	
H8W	0.2054	0.4183	0.7270	0.184*	
H7W	0.2016	0.2855	0.7659	0.184*	

### Atomic displacement parameters (Å^2^)

**Table:**

	*U*^11^	*U*^22^	*U*^33^	*U*^12^	*U*^13^	*U*^23^
C1	0.033 (2)	0.0229 (19)	0.029 (2)	−0.0026 (16)	−0.0122 (17)	−0.0030 (15)
Cl1	0.0593 (7)	0.0339 (6)	0.0312 (5)	−0.0140 (5)	0.0023 (5)	0.0009 (4)
N1	0.065 (3)	0.048 (2)	0.035 (2)	−0.011 (2)	−0.029 (2)	0.0090 (18)
Nd1	0.02726 (13)	0.01985 (12)	0.02122 (12)	−0.00926 (8)	−0.01255 (8)	0.00457 (8)
O1	0.0427 (17)	0.0241 (14)	0.0348 (15)	−0.0086 (12)	−0.0216 (13)	0.0045 (12)
C2	0.050 (3)	0.042 (3)	0.044 (3)	−0.018 (2)	−0.024 (2)	0.008 (2)
Cl2	0.0524 (7)	0.0347 (6)	0.0369 (6)	−0.0145 (5)	−0.0004 (5)	−0.0050 (4)
N2	0.041 (2)	0.045 (2)	0.0238 (17)	−0.0116 (17)	−0.0175 (15)	0.0086 (15)
O2	0.065 (2)	0.0323 (16)	0.0311 (16)	−0.0156 (15)	−0.0235 (15)	−0.0024 (13)
C3	0.062 (3)	0.066 (4)	0.052 (3)	−0.028 (3)	−0.038 (3)	0.011 (3)
O3	0.058 (2)	0.0298 (15)	0.0322 (15)	−0.0202 (14)	−0.0244 (14)	0.0116 (12)
C4	0.062 (3)	0.036 (2)	0.030 (2)	−0.011 (2)	−0.012 (2)	0.0079 (19)
N4	0.034 (2)	0.047 (2)	0.040 (2)	−0.0181 (17)	−0.0146 (16)	0.0039 (17)
O4	0.0366 (17)	0.0373 (17)	0.064 (2)	−0.0136 (14)	−0.0242 (16)	0.0137 (16)
C5	0.049 (3)	0.034 (2)	0.032 (2)	−0.012 (2)	−0.0127 (19)	0.0032 (18)
O5	0.0405 (18)	0.0414 (18)	0.059 (2)	−0.0164 (15)	−0.0250 (16)	0.0149 (15)
C6	0.0240 (19)	0.027 (2)	0.0237 (19)	−0.0069 (15)	−0.0082 (15)	0.0011 (15)
O6	0.047 (2)	0.089 (3)	0.086 (3)	−0.046 (2)	−0.029 (2)	0.029 (2)
C7	0.0282 (19)	0.0228 (18)	0.0221 (18)	−0.0047 (15)	−0.0090 (15)	0.0001 (14)
O7	0.0481 (18)	0.0371 (16)	0.0299 (15)	−0.0207 (14)	−0.0227 (13)	0.0041 (12)
C8	0.038 (2)	0.037 (2)	0.032 (2)	−0.0184 (19)	−0.0124 (18)	0.0051 (17)
O8	0.310 (11)	0.075 (4)	0.046 (3)	−0.008 (5)	−0.060 (4)	−0.001 (3)
C9	0.041 (2)	0.046 (3)	0.034 (2)	−0.018 (2)	−0.0219 (19)	0.0064 (19)
O9	0.056 (2)	0.070 (3)	0.056 (2)	−0.003 (2)	−0.0086 (19)	−0.027 (2)
C10	0.046 (3)	0.037 (2)	0.031 (2)	−0.018 (2)	−0.0105 (19)	0.0115 (18)
O10	0.101 (3)	0.041 (2)	0.057 (2)	−0.016 (2)	0.005 (2)	0.0161 (18)
C11	0.038 (2)	0.034 (2)	0.031 (2)	−0.0159 (18)	−0.0149 (18)	0.0057 (17)
O11	0.071 (4)	0.115 (5)	0.352 (13)	−0.052 (4)	0.074 (6)	−0.117 (7)
C12	0.030 (2)	0.0241 (19)	0.0229 (18)	−0.0074 (16)	−0.0102 (15)	0.0022 (15)
O12	0.367 (14)	0.100 (5)	0.062 (3)	0.058 (6)	−0.084 (6)	−0.037 (3)
O13	0.056 (2)	0.072 (3)	0.058 (2)	0.002 (2)	−0.0162 (19)	−0.030 (2)
O14	0.131 (5)	0.053 (3)	0.093 (4)	0.002 (3)	0.023 (3)	0.027 (3)
O15	0.085 (5)	0.160 (7)	0.55 (2)	−0.082 (5)	0.127 (8)	−0.224 (11)
O1W	0.0390 (17)	0.058 (2)	0.0377 (17)	−0.0217 (15)	−0.0107 (14)	−0.0038 (15)
O2W	0.0407 (17)	0.0286 (15)	0.0522 (19)	−0.0082 (13)	−0.0218 (15)	0.0024 (13)
O3W	0.056 (2)	0.0402 (18)	0.0296 (16)	−0.0151 (15)	−0.0006 (14)	0.0030 (13)
O4W	0.143 (6)	0.133 (6)	0.088 (4)	−0.048 (5)	0.007 (4)	−0.009 (4)

### Geometric parameters (Å, °)

**Table:**

C1—C2	1.382 (6)	O2—C6	1.240 (5)
C1—C5	1.385 (6)	O2—Nd1^i^	2.392 (3)
C1—C6	1.506 (5)	C3—H3	0.9300
Cl1—O11	1.367 (6)	O3—C12	1.245 (5)
Cl1—O10	1.405 (4)	C4—C5	1.372 (6)
Cl1—O8	1.408 (6)	C4—H4	0.9300
Cl1—O9	1.413 (4)	N4—O6	1.220 (5)
N1—C4	1.321 (7)	N4—O5	1.250 (5)
N1—C3	1.324 (7)	N4—O4	1.275 (5)
N1—H1	0.8600	C5—H5	0.9300
Nd1—O1	2.428 (3)	C7—C11	1.383 (6)
Nd1—O2^i^	2.392 (3)	C7—C8	1.389 (6)
Nd1—O3	2.446 (3)	C7—C12	1.512 (5)
Nd1—O4	2.571 (3)	C8—C9	1.365 (6)
Nd1—O5	2.651 (3)	C8—H8	0.9300
Nd1—O7^ii^	2.390 (3)	C9—H9	0.9300
Nd1—O1W	2.542 (3)	C10—C11	1.371 (6)
Nd1—O2W	2.567 (3)	C10—H10	0.9300
Nd1—O3W	2.505 (3)	C11—H11	0.9300
O1—C6	1.245 (5)	C12—O7	1.242 (5)
C2—C3	1.379 (7)	O1W—H1W	0.8400
C2—H2	0.9300	O1W—H2W	0.8400
Cl2—O15	1.311 (6)	O2W—H4W	0.8400
Cl2—O12	1.379 (6)	O2W—H3W	0.8400
Cl2—O14	1.388 (5)	O3W—H6W	0.8400
Cl2—O13	1.417 (4)	O3W—H5W	0.8400
N2—C10	1.329 (6)	O4W—H8W	0.8400
N2—C9	1.339 (6)	O4W—H7W	0.8400
N2—H2A	0.8600		
			
C2—C1—C5	119.2 (4)	O12—Cl2—O14	104.1 (5)
C2—C1—C6	120.9 (4)	O15—Cl2—O13	110.6 (4)
C5—C1—C6	119.9 (4)	O12—Cl2—O13	108.2 (4)
O11—Cl1—O10	111.3 (4)	O14—Cl2—O13	113.7 (3)
O11—Cl1—O8	107.8 (6)	C10—N2—C9	122.9 (4)
O10—Cl1—O8	106.1 (3)	C10—N2—H2A	118.6
O11—Cl1—O9	110.7 (4)	C9—N2—H2A	118.6
O10—Cl1—O9	113.1 (3)	C6—O2—Nd1^i^	162.7 (3)
O8—Cl1—O9	107.4 (4)	N1—C3—C2	120.4 (5)
C4—N1—C3	122.6 (4)	N1—C3—H3	119.8
C4—N1—H1	118.7	C2—C3—H3	119.8
C3—N1—H1	118.7	C12—O3—Nd1	134.0 (3)
O7^ii^—Nd1—O2^i^	140.53 (12)	N1—C4—C5	119.6 (5)
O7^ii^—Nd1—O1	81.57 (10)	N1—C4—H4	120.2
O2^i^—Nd1—O1	85.82 (10)	C5—C4—H4	120.2
O7^ii^—Nd1—O3	97.66 (10)	O6—N4—O5	122.0 (4)
O2^i^—Nd1—O3	75.38 (10)	O6—N4—O4	121.0 (4)
O1—Nd1—O3	149.37 (11)	O5—N4—O4	117.0 (4)
O7^ii^—Nd1—O3W	75.54 (11)	N4—O4—Nd1	98.8 (2)
O2^i^—Nd1—O3W	136.17 (11)	C4—C5—C1	119.5 (4)
O1—Nd1—O3W	74.83 (10)	C4—C5—H5	120.2
O3—Nd1—O3W	134.93 (11)	C1—C5—H5	120.2
O7^ii^—Nd1—O1W	69.63 (11)	N4—O5—Nd1	95.6 (2)
O2^i^—Nd1—O1W	70.93 (11)	O2—C6—O1	127.0 (4)
O1—Nd1—O1W	73.20 (11)	O2—C6—C1	116.9 (3)
O3—Nd1—O1W	77.83 (11)	O1—C6—C1	116.1 (3)
O3W—Nd1—O1W	135.33 (11)	C11—C7—C8	119.0 (4)
O7^ii^—Nd1—O2W	70.39 (10)	C11—C7—C12	120.4 (4)
O2^i^—Nd1—O2W	137.28 (10)	C8—C7—C12	120.5 (3)
O1—Nd1—O2W	135.29 (10)	C9—C8—C7	119.4 (4)
O3—Nd1—O2W	70.71 (10)	C9—C8—H8	120.3
O3W—Nd1—O2W	65.02 (10)	C7—C8—H8	120.3
O1W—Nd1—O2W	124.20 (11)	N2—C9—C8	119.5 (4)
O7^ii^—Nd1—O4	139.99 (11)	N2—C9—H9	120.2
O2^i^—Nd1—O4	77.38 (12)	C8—C9—H9	120.2
O1—Nd1—O4	122.18 (10)	N2—C10—C11	119.5 (4)
O3—Nd1—O4	77.44 (10)	N2—C10—H10	120.3
O3W—Nd1—O4	80.43 (12)	C11—C10—H10	120.3
O1W—Nd1—O4	143.71 (11)	C10—C11—C7	119.6 (4)
O2W—Nd1—O4	70.58 (10)	C10—C11—H11	120.2
O7^ii^—Nd1—O5	138.71 (10)	C7—C11—H11	120.2
O2^i^—Nd1—O5	70.41 (12)	O7—C12—O3	126.1 (3)
O1—Nd1—O5	73.54 (10)	O7—C12—C7	117.5 (3)
O3—Nd1—O5	120.41 (10)	O3—C12—C7	116.4 (3)
O3W—Nd1—O5	66.52 (11)	Nd1—O1W—H1W	115.1
O1W—Nd1—O5	130.00 (11)	Nd1—O1W—H2W	114.5
O2W—Nd1—O5	105.70 (11)	H1W—O1W—H2W	111.6
O4—Nd1—O5	48.66 (10)	Nd1—O2W—H4W	120.0
C6—O1—Nd1	140.8 (3)	Nd1—O2W—H3W	112.9
C3—C2—C1	118.4 (5)	H4W—O2W—H3W	111.4
C3—C2—H2	120.8	Nd1—O3W—H6W	127.8
C1—C2—H2	120.8	Nd1—O3W—H5W	119.0
O15—Cl2—O12	107.9 (8)	H6W—O3W—H5W	111.3
O15—Cl2—O14	111.9 (6)	H8W—O4W—H7W	111.5

Symmetry codes: (i) −*x*+1, −*y*+1, −*z*+1; (ii) −*x*+1, −*y*, −*z*+1.

### Hydrogen-bond geometry (Å, °)

**Table:**

*D*—H···*A*	*D*—H	H···*A*	*D*···*A*	*D*—H···*A*
O4W—H7W···O12	0.84	2.59	3.256 (11)	136
O4W—H8W···O5^i^	0.84	2.52	3.023 (8)	119
O4W—H8W···O8^i^	0.84	2.04	2.801 (9)	150
O3W—H5W···O12^ii^	0.84	2.11	2.945 (7)	170
O3W—H6W···O8	0.84	2.04	2.846 (8)	162
O2W—H3W···O1W^ii^	0.84	2.12	2.900 (5)	154
O2W—H4W···O4^iii^	0.84	2.04	2.861 (4)	167
O1W—H2W···O4W	0.84	1.83	2.593 (7)	150
O1W—H1W···O6^iv^	0.84	2.04	2.880 (5)	178
N2—H2A···O9^iii^	0.86	2.51	3.045 (5)	121
N2—H2A···O13^v^	0.86	2.48	3.033 (5)	123
N2—H2A···O10^vi^	0.86	2.15	2.868 (5)	141
N1—H1···O13^vii^	0.86	2.46	2.994 (6)	121
N1—H1···O9^viii^	0.86	2.46	2.988 (6)	120
N1—H1···O14^ix^	0.86	2.24	2.953 (6)	140

Symmetry codes: (i) −*x*+1, −*y*+1, −*z*+1; (ii) −*x*+1, −*y*, −*z*+1; (iii) −*x*+2, −*y*, −*z*+1; (iv) *x*−1, *y*, *z*; (v) −*x*+1, −*y*, −*z*+2; (vi) *x*, *y*−1, *z*+1; (vii) −*x*, −*y*+1, −*z*+1; (viii) −*x*+1, −*y*+1, −*z*; (ix) *x*, *y*+1, *z*−1.
